# Supplementary material for: Transient IGF-1R inhibition combined with osimertinib eradicates AXL-low expressing EGFR mutated lung cancer
Source: Nat Commun. 2020 Sep 14;11:4607. doi: 10.1038/s41467-020-18442-4 (PMC7490421; doi:10.1038/s41467-020-18442-4)
Supplement: Supplementary file 1 — Supplementary Information [file 41467_2020_18442_MOESM1_ESM.pdf]

Supplementary Figure 1

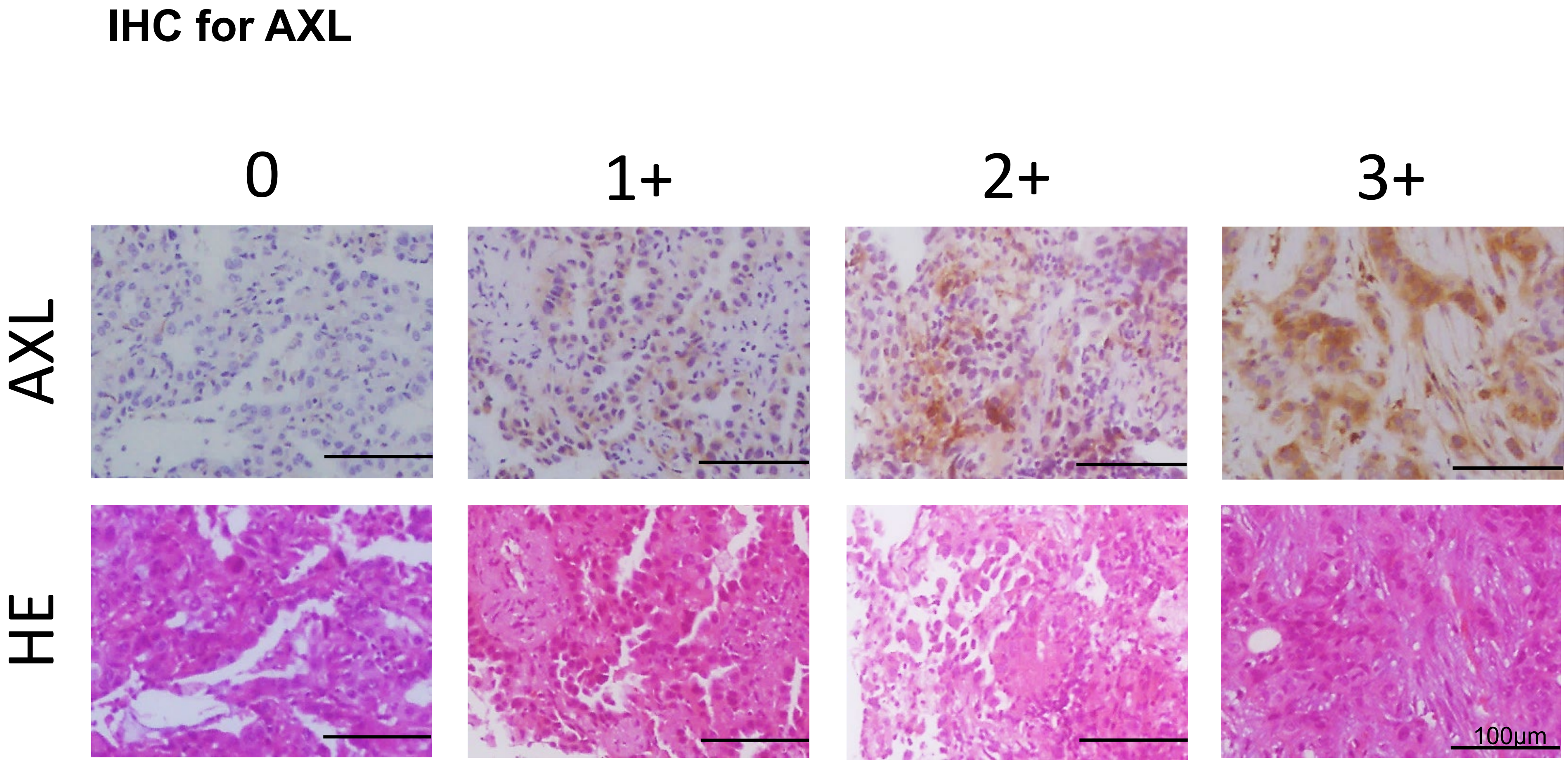

Supplementary Figure 1. Representative results of immunohistochemical staining for AXL expression. Bar, 100 μm.

Supplementary Figure 2

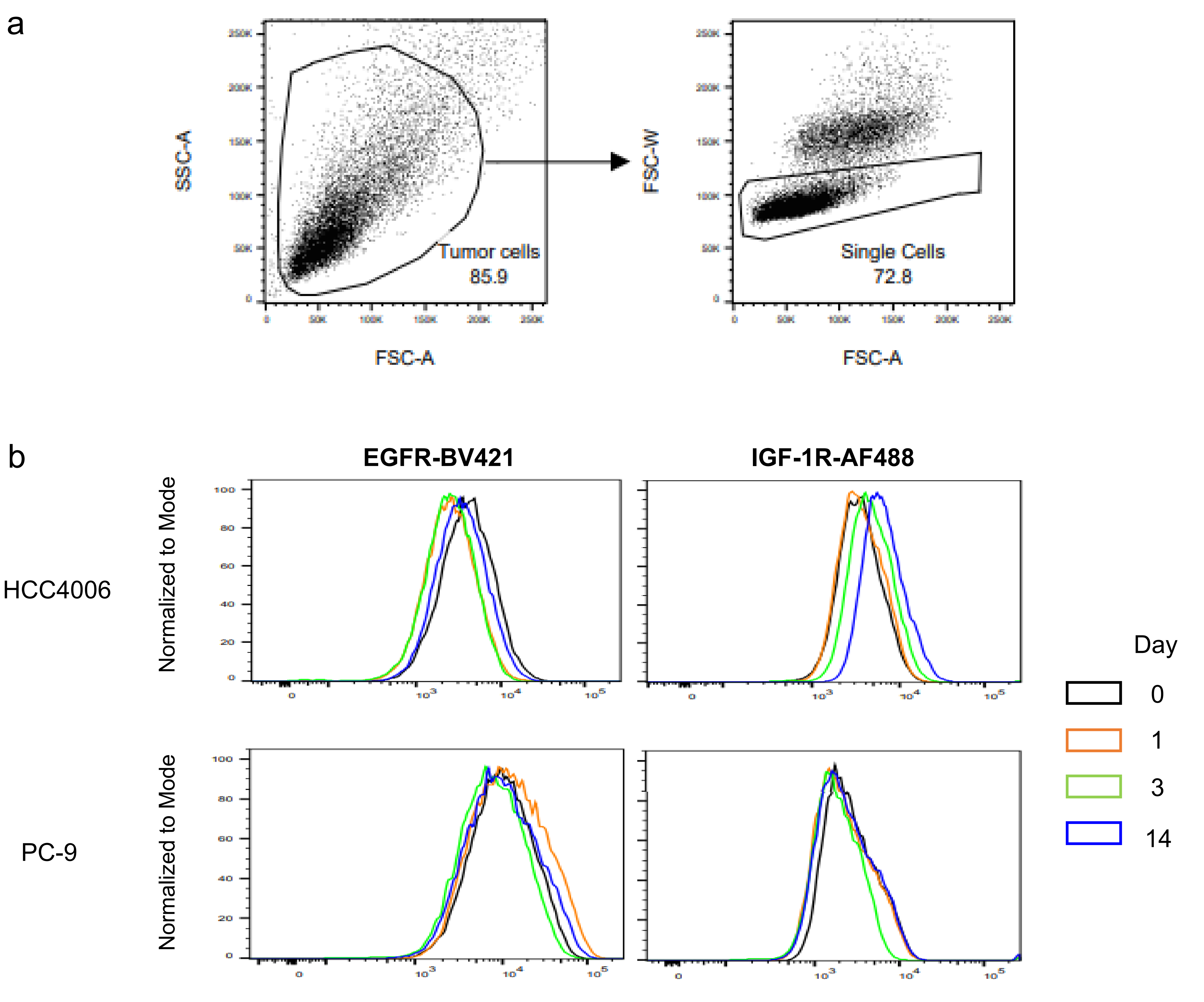

c

Mean fluorescence intensity (MFI)

Day	HCC4006 IGF-1R	HCC4006 EGFR	PC-9 IGF-1R	PC-9 EGFR
0	4087	5032	3858	13643
1	4313	3135	3652	18752
3	5690	3017	2788	10605
14	7697	4219	3945	15045

Supplementary Figure 2. Expression of IGF-1R and EGFR in osimertinib treated *EGFR* mutated NSCLC cell lines determined by flow cytometry. a Tumor cell lines were gated on single cell gates followed by FSC-A/SSC-A tumor cells population. **b** HCC4006 and PC-9 cells were treated with osimertinib (0.3 µmol/L and 3 µmol/L, respectively) for 14 days. At indicated time points, the cells were harvested and treated with anti-EGFR or anti-IGF-1R antibody for 30 min on ice. Then, the cells were washed three times and treated with a secondary antibody for 30 min on ice. The protein expression was determined by flow cytometry. **c** The mean fluorescence intensity is shown. Data shown are representative of three independent experiments.

Supplementary Figure 3

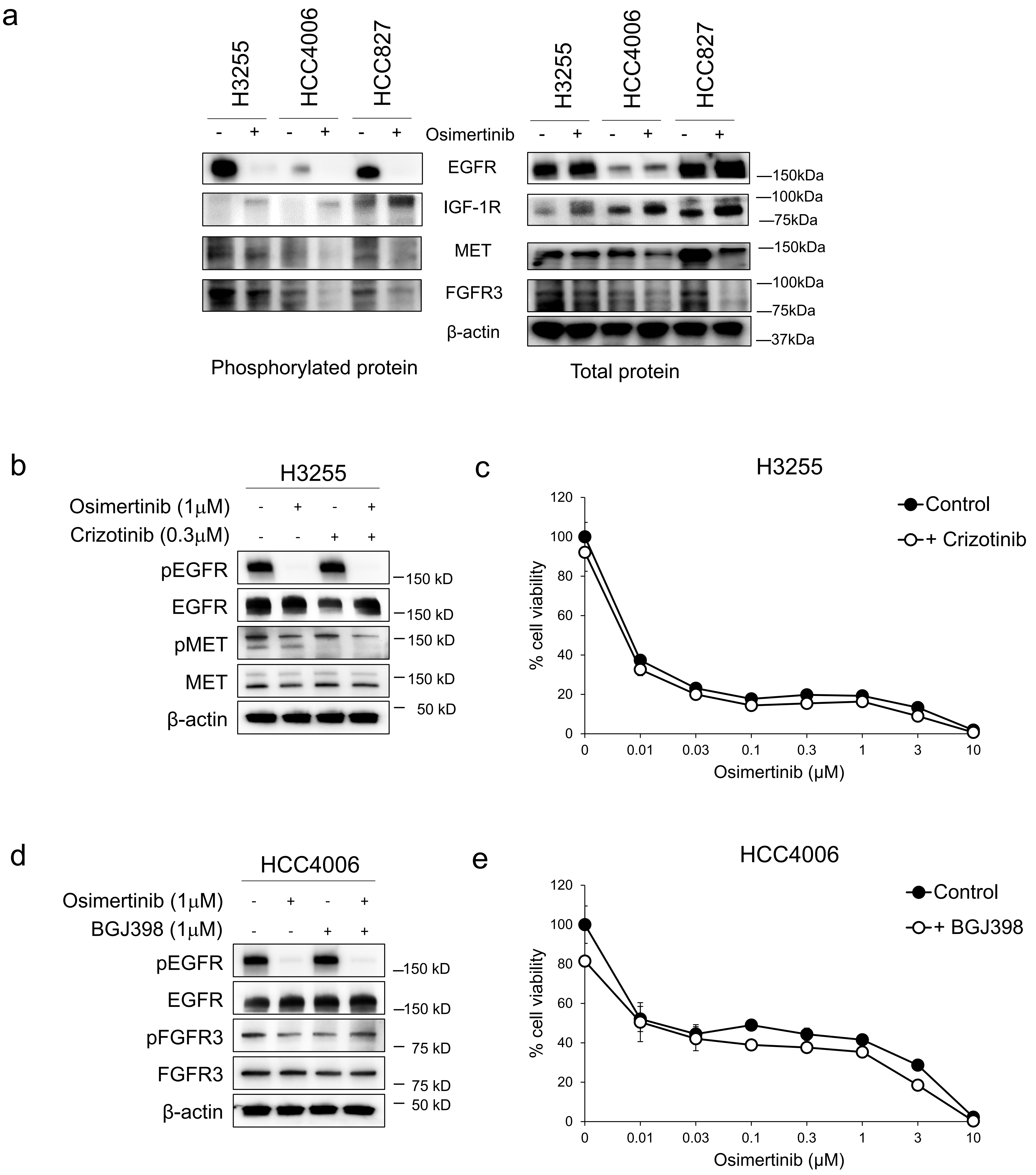

Supplementary Figure 3. Role of MET and FGFR3 on sensitivity to osimertinib in H3255 and HCC4006 cells, respectively. a *EGFR* mutated NSCLC cell lines were treated with osimertinib (30 nmol/L for HCC4006 and H3255 cells, 300 nmol/L for HCC827 cells) for 72 h, and lysates were analyzed by western blotting. **b** H3255 cells were treated with osimertinib and/or crizotinib (a MET inhibitor) for 1 h, and lysates were analyzed by western blotting. **c** H3255 cells were treated with osimertinib and/or crizotinib (0.3μmol/L) for 72 h, and cell viability was determined by MTT assay. **d** HCC4006 cells were treated with osimertinib and/or BJG398 (a FGFR inhibitor) for 1 h, and lysates were analyzed by western blotting. **e** HCC4006 cells were treated with osimertinib and/or BJG398 (1μmol/L) for 72 h, and cell viability was determined by MTT assay. Data shown are representative of three independent experiments. Data are represented as mean ± s.d. Bars showed s.d.

Supplementary Figure 4

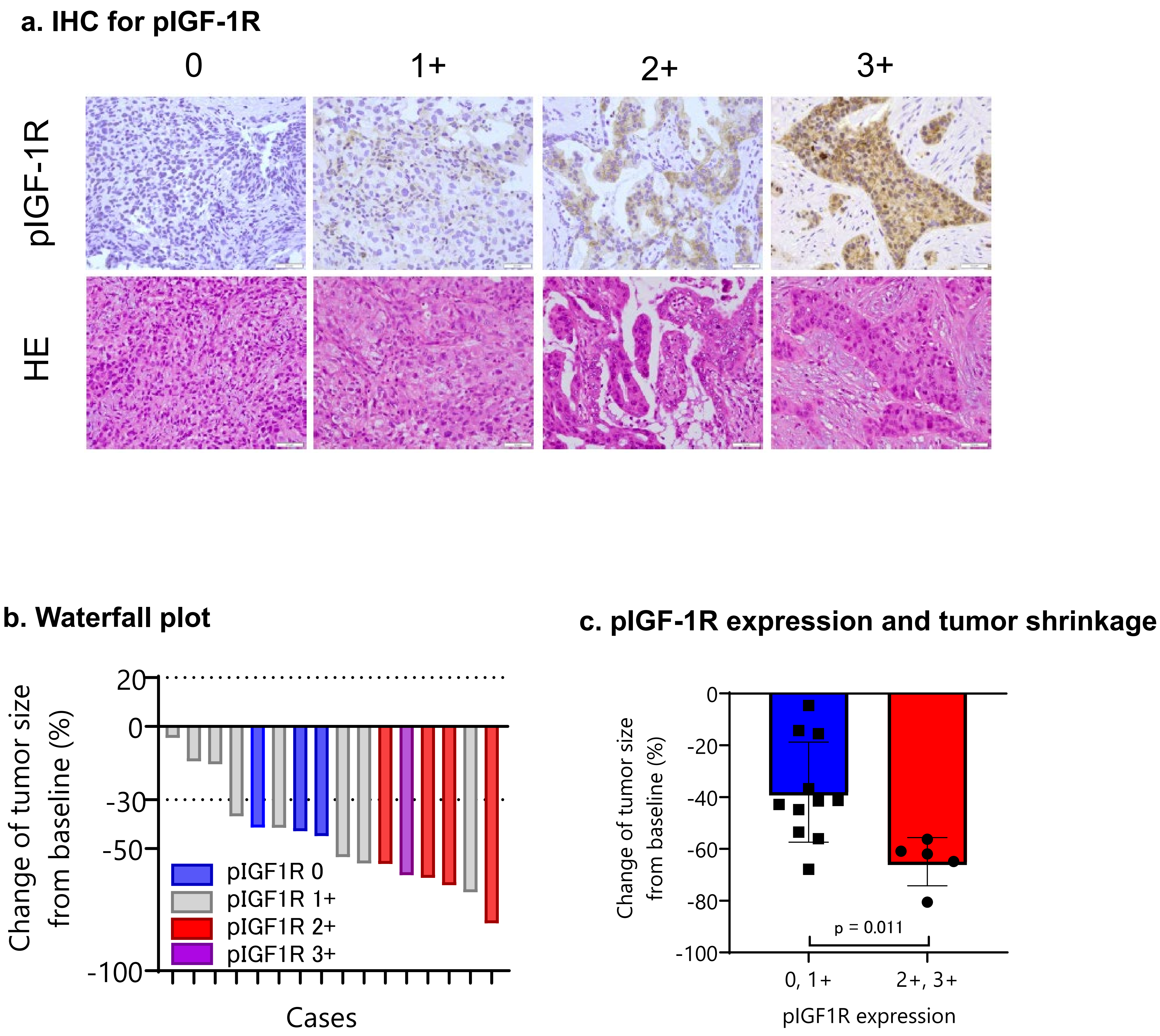

Supplementary Figure 4. pIGF-1R expression correlates with osimertinib sensitivity in AXL-low expressing *EGFR* mutated NSCLC patients. a Representative results of immunohistochemical staining for pIGF-1R expression. Bar, 100 μm. **b** Correlation between the pIGF-1Rexpression, determine by IHC, and the response to osimertinib treatment in AXL-low expressing *EGFR* mutated NSCLC specimens from 16 patients. **c** Change of tumor size from baseline following osimertinib treatment in *EGFR* mutated NSCLC patients with low (no to 1+)-pIGF-1R expression (N=11) and high (2+ to 3+)-pIGF-1R expression (N=5). The data are expressed as mean and SDs. P=0.011 compared by two-sided Student’s t-tests.

Supplementary Figure 5

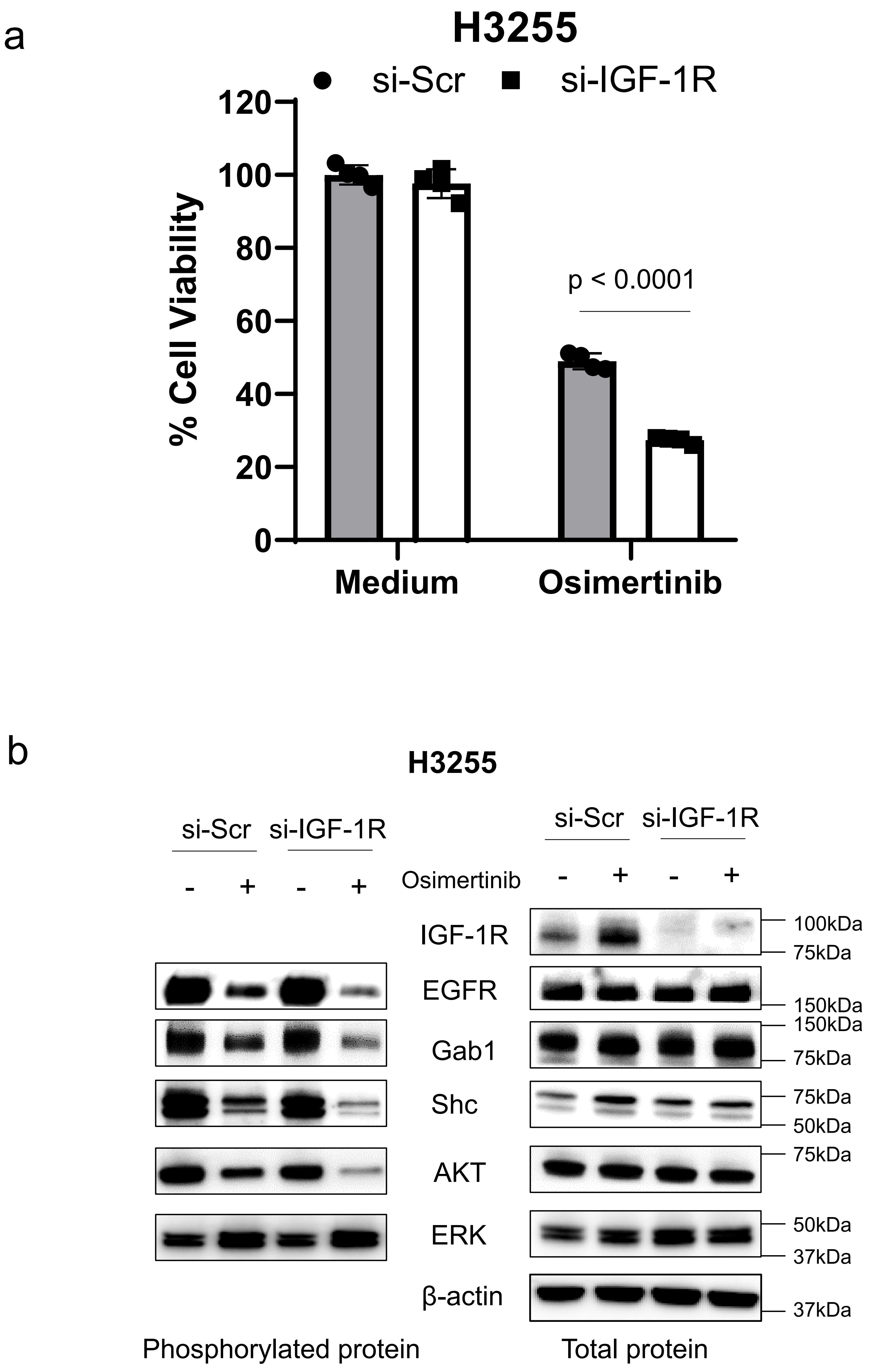

Supplementary Figure 5. Knockdown of IGF-1R decreases the viability of H3255 cells. **a** H3255 cells were treated with si-Scr or si-IGF-1R for 72 h in the presence or absence of osimertinib (30 nmol/L), and cell viability was determined. The percentage of growth is shown relative to untreated controls. Each sample was assayed in triplicate, with each experiment repeated at least three times independently. p value is provided (one-way ANOVA). Data are represented as mean ± s.d. Bars showed s.d. **b** si-Scr or IGF-1R-specific siRNA was introduced into H3255 cells. After 24 h, the cells were incubated with or without osimertinib (30 nmol/L) for 72 h and lysed, and the indicated proteins were detected by western blotting. Data shown are representative of three independent experiments.

Supplementary Figure 6

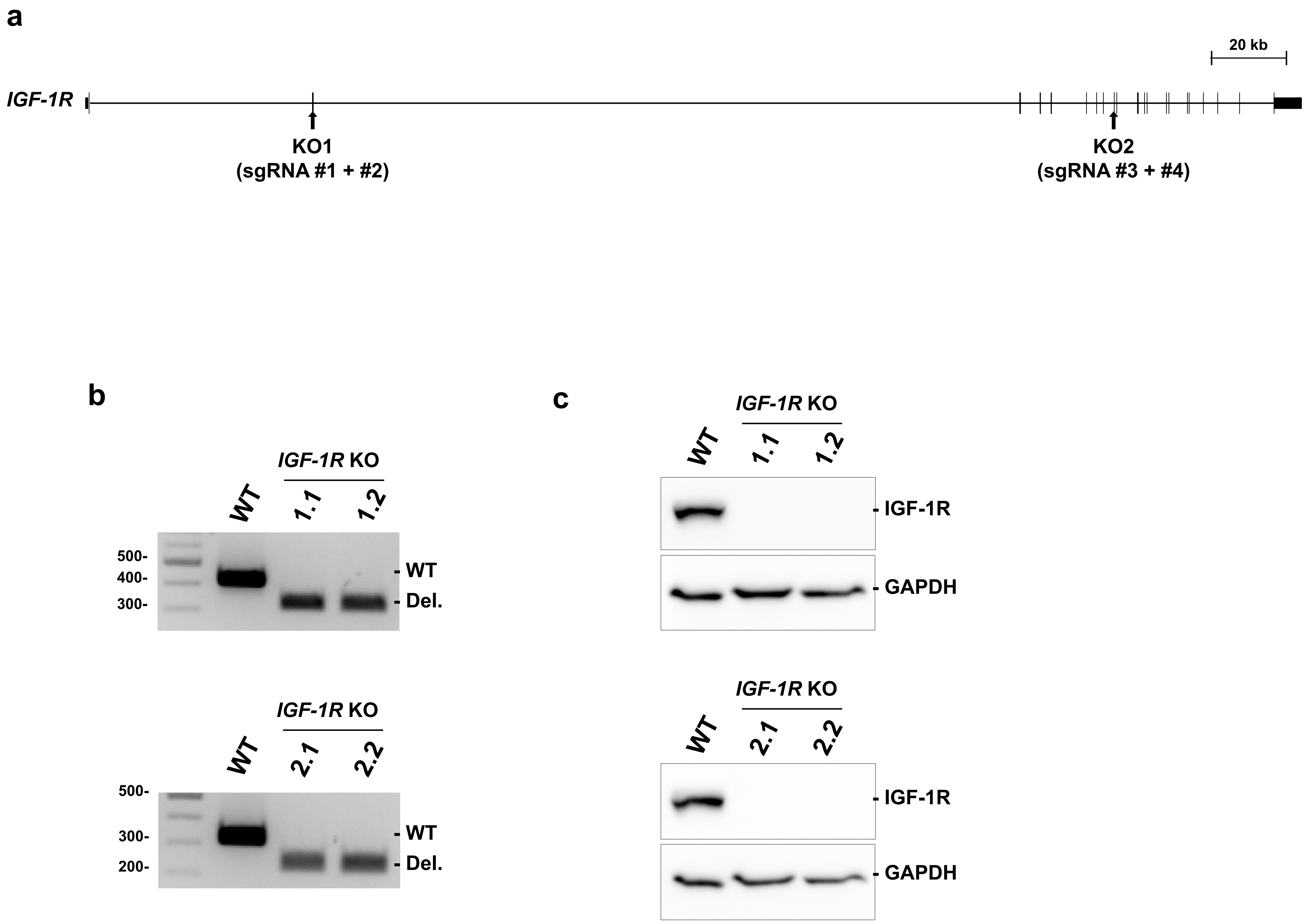

Supplementary Figure 6. Generation of *IGF1R* knockout HCC827 cells. a The location of DNA sequences corresponding to the *IGF1R*-specific sgRNAs for creating two kinds of knockout cells, KO1 and KO2. Co-expression of sgRNA #1 and #2 can partially delete exon 2 in *IGF-1R* locus, whereas co-expression of sgRNA #3 and #4 can partially delete exon 9. **b** PCR analysis with genomic DNAs from wild-type (WT) HCC827, two independent clones of KO1 (1.1 and 1.2) and KO2 (2.1 and 2.2) *IGF-1R*-knockout cells. Note that homologous deletion was observed in both KO1 and KO2 cells. **c** Western blotting of WT HCC827 and *IGF-1R*-knockout cell clones with anti-IGF-1R antibody. GAPDH was used as a loading control. Data shown are representative of three independent experiments.

Supplementary Figure 7

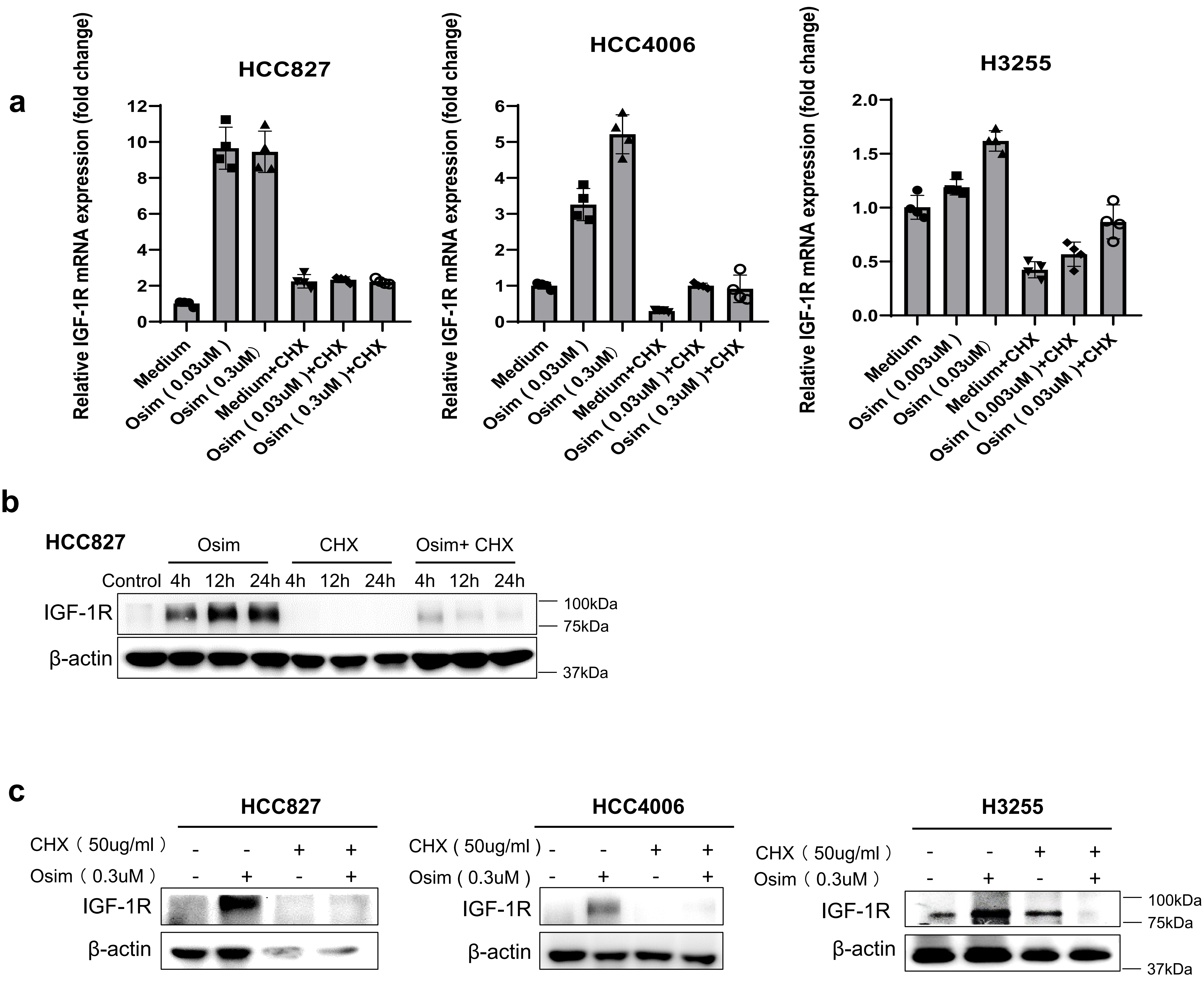

Supplementary Figure 7. Effect of CHX on IGF-1R expression. a Tumor cells were treated with osimertinib and or cycloheximide (CHX) (50 mg/ml) for 24 h. The mRNA was harvested and the IGF-1R mRNA expression was evaluated by qRT-PCR. Data are represented as mean ± s.d. Bars showed s.d. Each sample was assayed in triplicate, with each experiment repeated at least three times independently. **b** HCC827 cells were treated with osimertinib (0.3 mmol/L) and or CHX (50 mg/ml) for indicated periods. Then, lysates were harvested and assessed by western blot. **c** Tumor cells were treated with osimertinib (0.3 mmol/L) and or CHX (50 mg/ml) for 24 h. Next, lysates were harvested and assessed by western blotting. Data shown are representative of three independent experiments.

Supplementary Figure 8

a

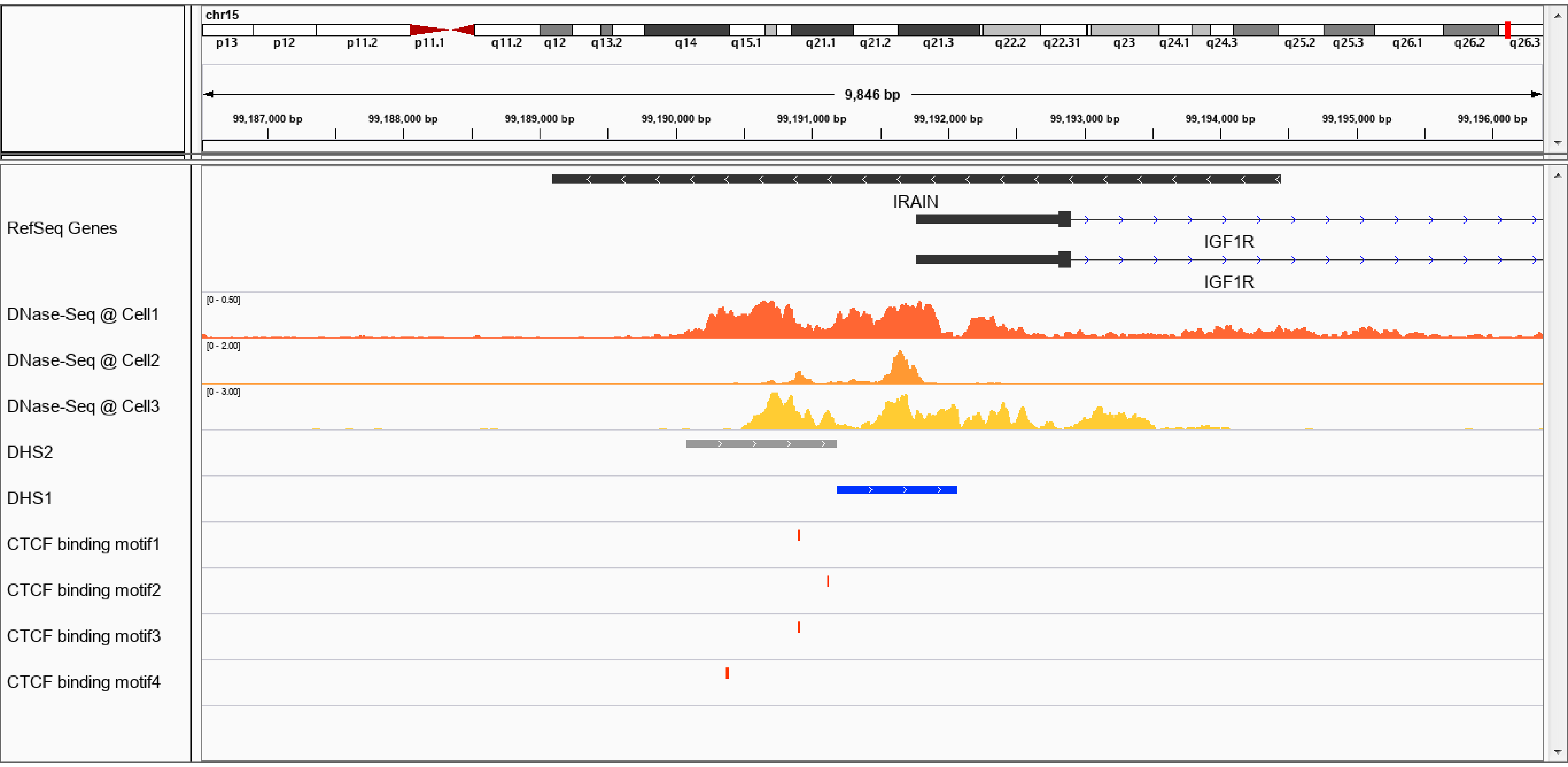

b

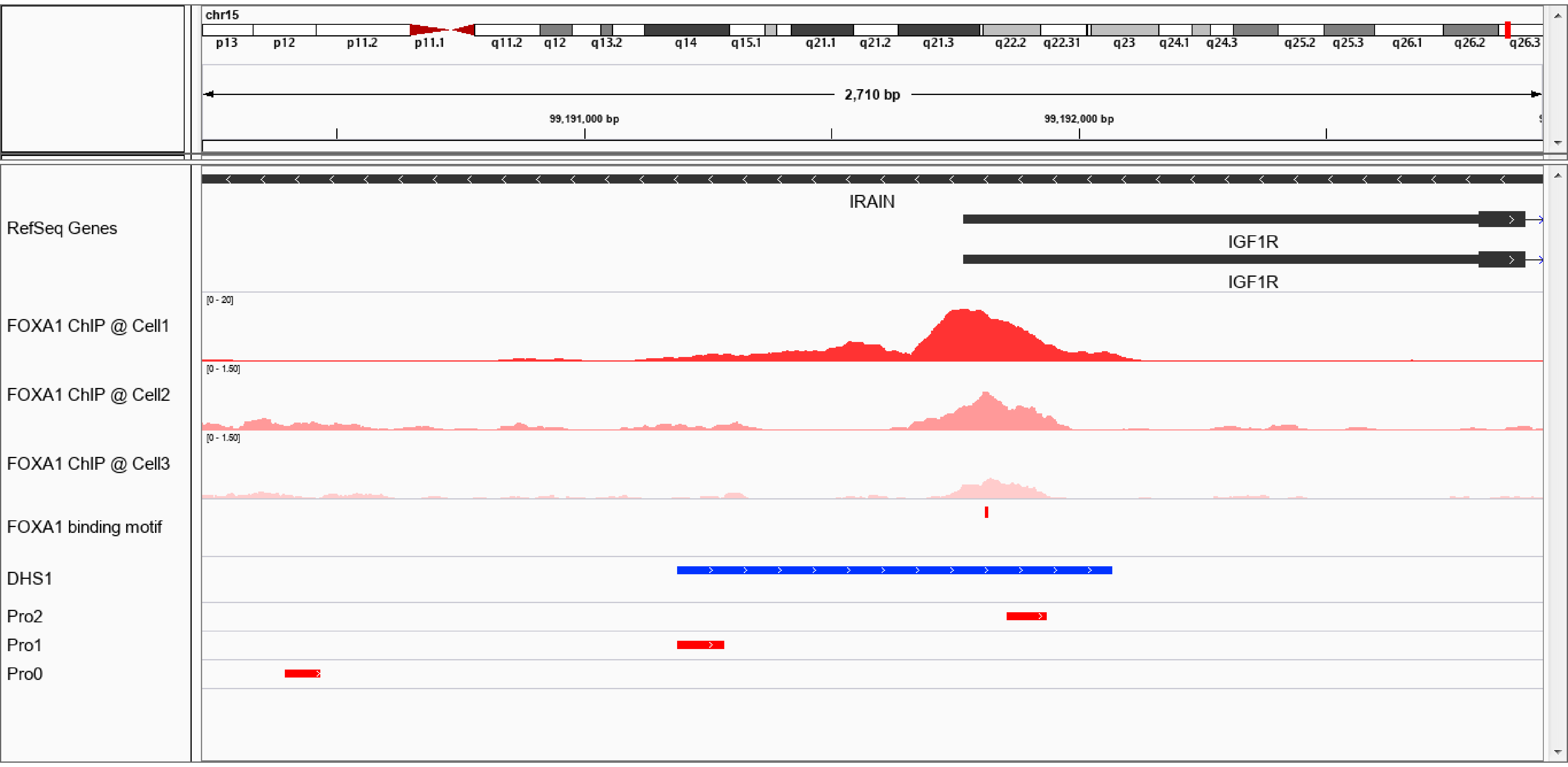

Supplementary Figure 8. The binding motif of the FOXA1 transcription factor is located within the DNase I hypersensitivity sites (DHS) region in the IGF-1R gene. a Visualization of DNase sensitive peaks, predicted DHSs, and CTCF binding motifs around IGF-1R TSS. We first visualized public DNase1 sensitive peaks by ChIP-Atlas (Oki et al., 2018) and then defined DNase sensitive peaks enriched regions as DHS1 (blue bar) and DHS2 (grey bar). Representative three DNase sensitive peaks are shown in orange. Four CTCF insulator binding motifs were shown with red boxes. **b** Visualization of three FOXA1 ChIP-seq peaks (red peaks), FOXA1 binding motif (small red bar), DHS1 (blue bar) and ChIP-qPCR amplified regions (red bars) around IGF-1R TSS.

Supplementary Figure 9

a *EGFR* mutated NSCLC cells produced low level of IGF-1 and IGF-2

Cell line	IGF-1 (ng/ml)	IGF-2 (ng/ml)
HCC4006	0.23	1.29
HCC827	0.27	0.05
H3255	0.27	0.57
PC-9	0.52	0.42
PC-9/GXR	0.86	0.37
HCC4011	0.68	0.26

b Exogenously added IGF-1 activated IGF-1R in *EGFR* mutated NSCLC cells

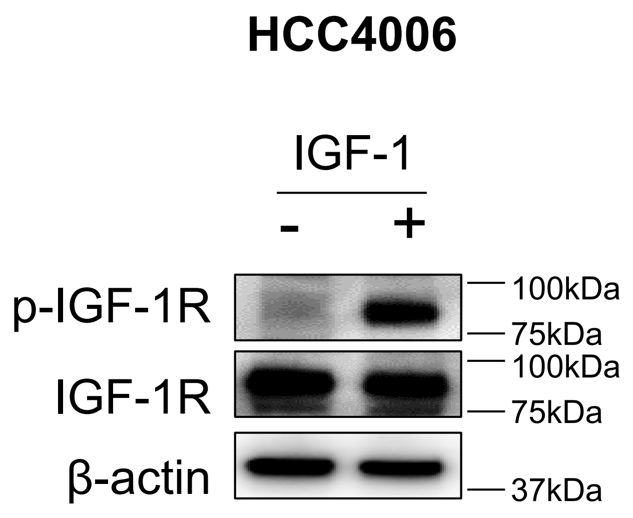

c Exogenously added IGF-1 did not affect osimertinib sensitivity of *EGFR* mutated NSCLC cell lines

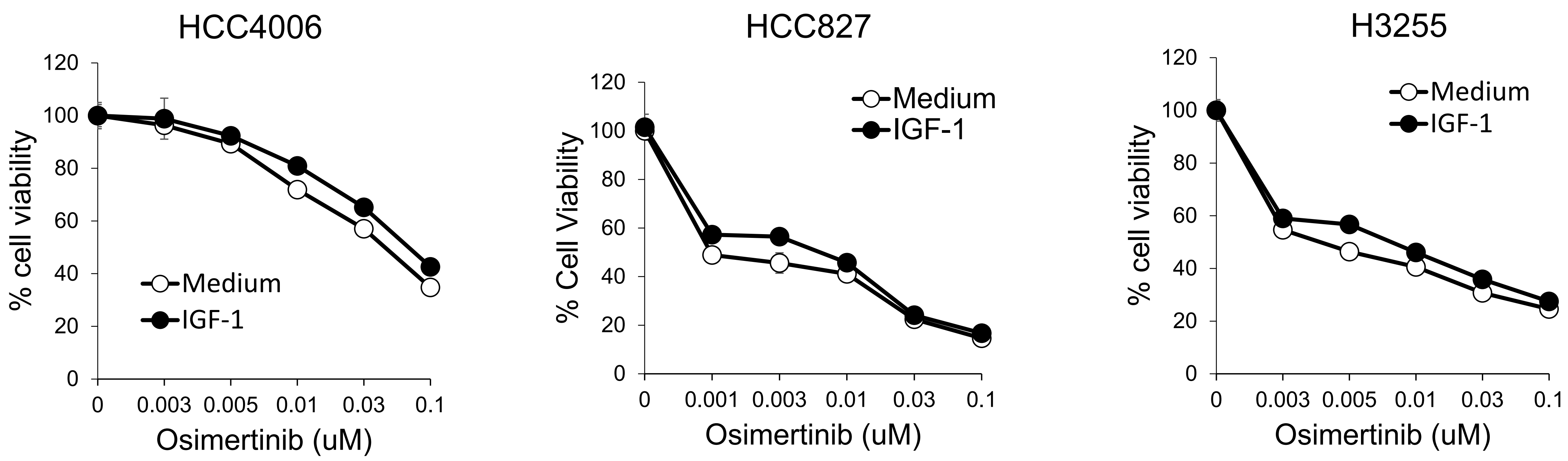

Supplementary Figure 9. IGF-1R ligand expression by *EGFR* mutated NSCLC cell lines. a *EGFR* mutated NSCLC cells (10^5 cells/2 ml in a 6-well plate) were incubated for 48 h and culture supernatants were harvested. The level of IGF-1 in the supernatants was determined by ELISA. **b** HCC4006 cells were incubated with or without recombinant human IGF-1 (100 ng/ml). Then, the lysates were harvested and evaluated by western blotting. Data shown are representative of three independent experiments. **c** HCC4006, HCC827, and H3255 cells were incubated with various concentrations of osimertinib for 72 h, in the presence or absence of recombinant human IGF-1 (100 ng/ml) and cell viability was determined. The percentage of growth is shown relative to untreated controls. Data are represented as mean ± s.d. Bars showed s.d. Each sample was assayed in triplicate, with each experiment repeated at least three times independently.

Supplementary Figure 10

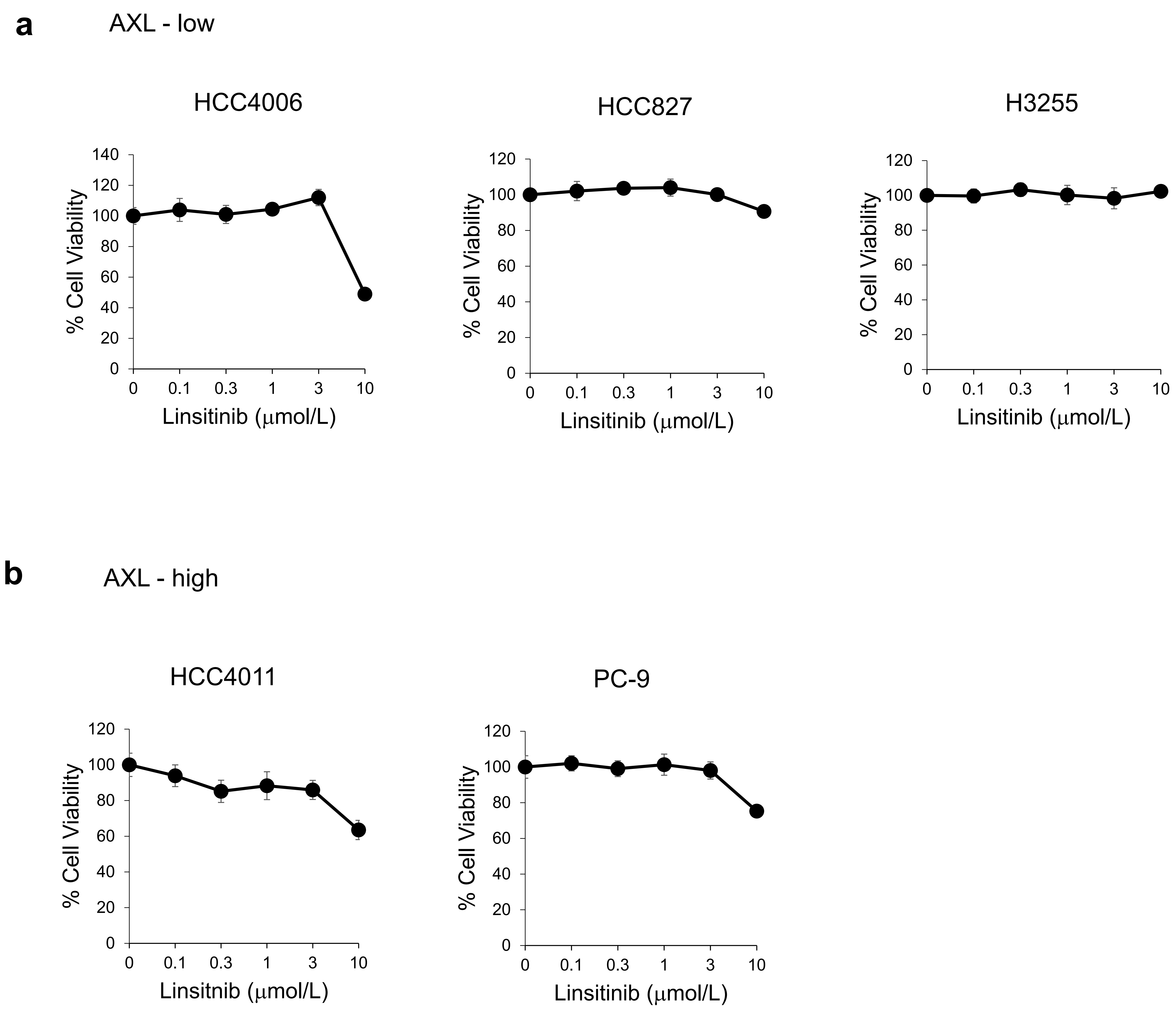

Supplementary Figure 10. Linsitinib does not affect the viability of *EGFR* mutated NSCLC cell lines. AXL-low expressing **(a)** and AXL-high expressing **(b)** *EGFR* mutated NSCLC cell lines were treated with various concentrations of linsitinib for 72 h, and cell viability was determined. The percentage of growth is shown relative to untreated controls**.** Data are represented as mean ± s.d. Bars showed s.d. Each sample was assayed in triplicate, with each experiment repeated at least three times independently.

Supplementary Figure 11

a

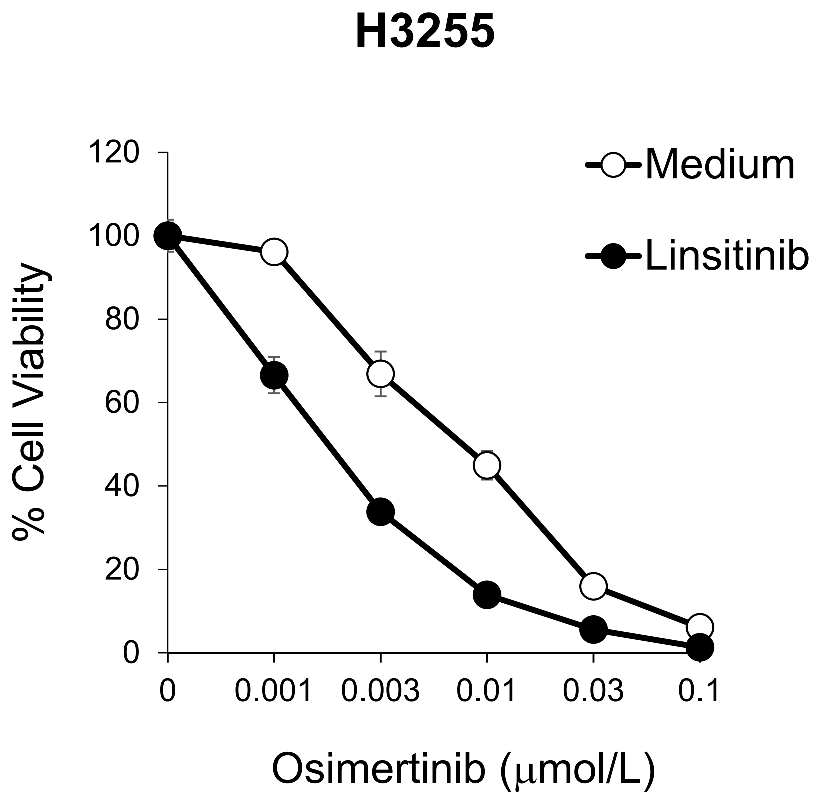

b

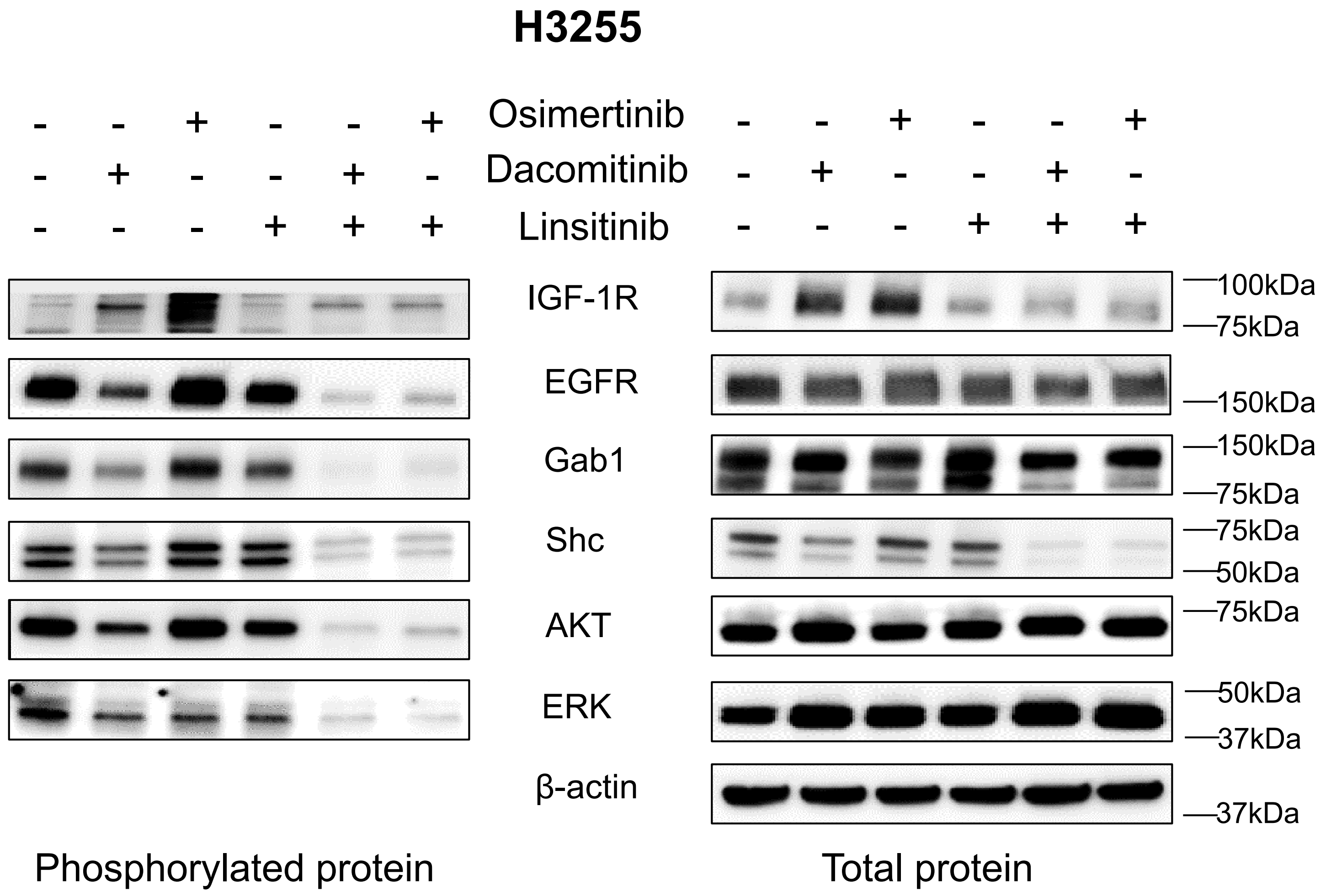

Supplementary Figure 11. Linsitinib inhibits the viability of H3255 cells exposed to osimertinib. **a** H3255 cells were treated with various concentrations of osimertinib for 72 h in the presence or absence of linsitinib (1 μmol/L), and cell viability was determined. The percentage of growth is shown relative to untreated controls. Data are represented as mean ± s.d. Bars showed s.d. Each sample was assayed in triplicate, with each experiment repeated at least three times independently. **b** H3255 cells were treated with osimertinib (30 nmol/L), dacomitinib (3 nmol/L), and or linsitinib (1 μmol/L). After 72 h, the cells were lysed, and the indicated proteins were detected by western blotting. Data shown are representative of three independent experiments.

Supplementary Figure 12

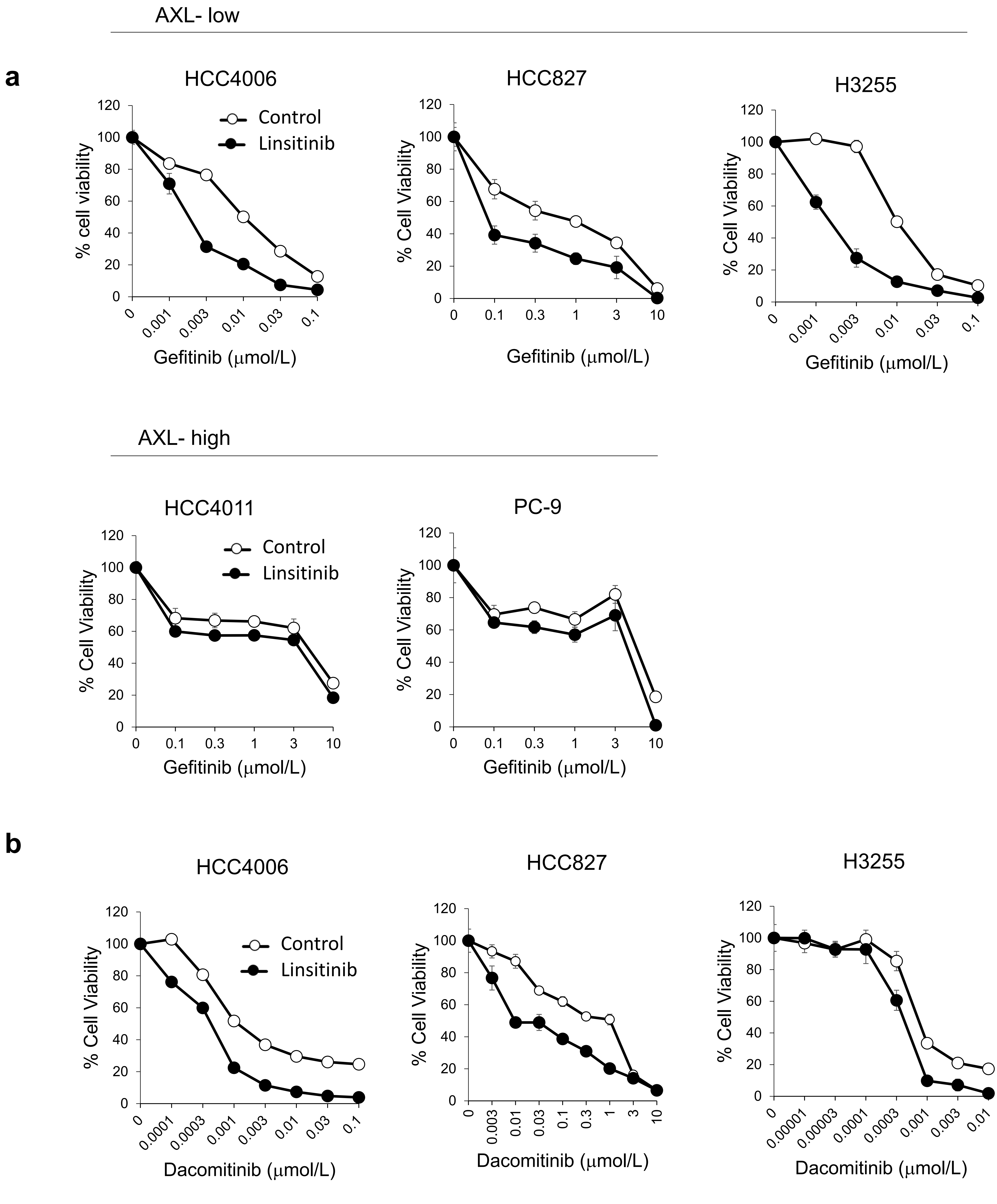

Supplementary Figure 12. Linsitinib inhibits the viability of AXL-low expressing *EGFR* mutated NSCLC cell lines exposed to gefitinib or dacomitinib. *EGFR* mutated NSCLC cell lines were treated with various concentrations of gefitinib **(a)** or dacomitinib **(b)** for 72 h in the presence or absence of linsitinib (1 μmol/L), and cell viability was determined. The percentage of growth is shown relative to untreated controls. Data are represented as mean ± s.d. Bars showed s.d. Each sample was assayed in triplicate, with each experiment repeated at least three times independently.

Supplementary Figure 13

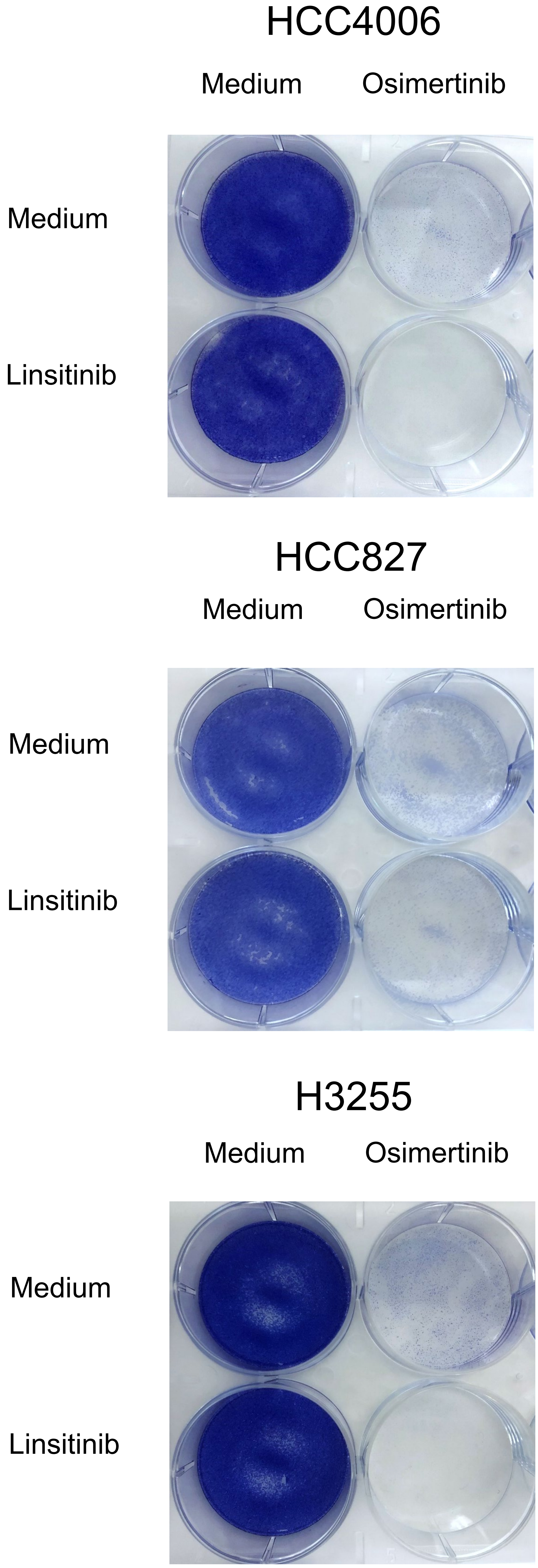

Supplementary Figure 13. Linsitinib and osimertinib cultured for 7 days inhibit the viability of AXL-low expressing *EGFR* mutated NSCLC cell lines. HCC4006, HCC827, and H3255 cells were cultured for 7 days in the presence or absence of linsitinib (1 μmol/L) and or osimertinib (500 nmol/L) in a 6-well plate. The plates were stained with crystal violet and imaged. A representative plate of three independent experiments is shown.

Supplementary Figure 14

a Body weight of HCC4006-inoculated mice treated with continuous combination

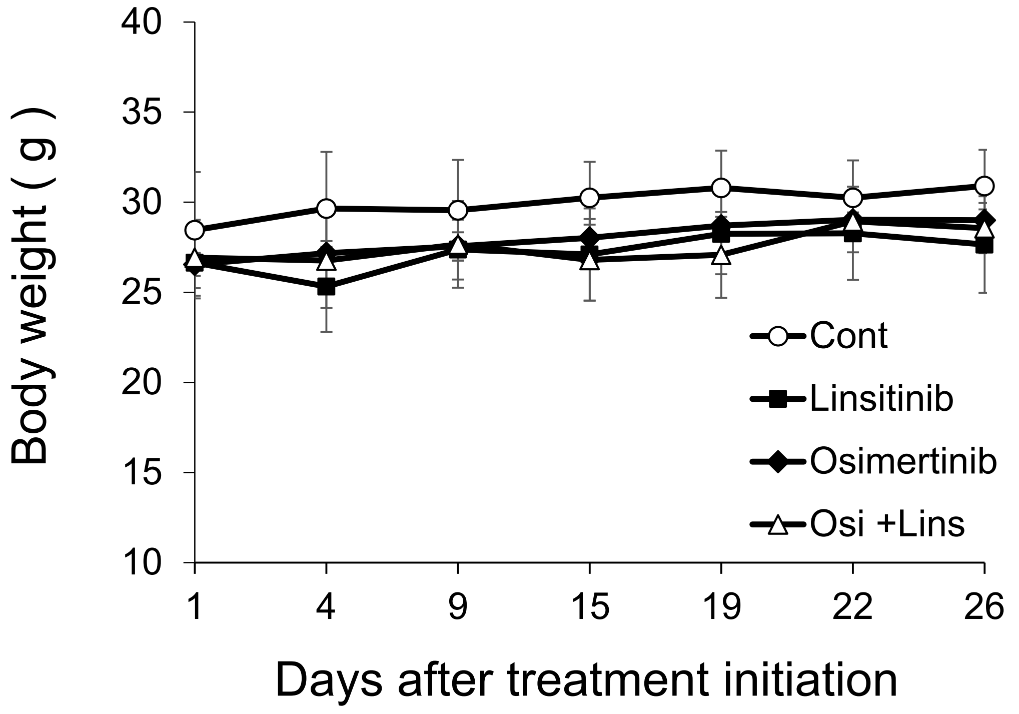

b Body weight of HCC4006-inoculated mice treated with transient combination

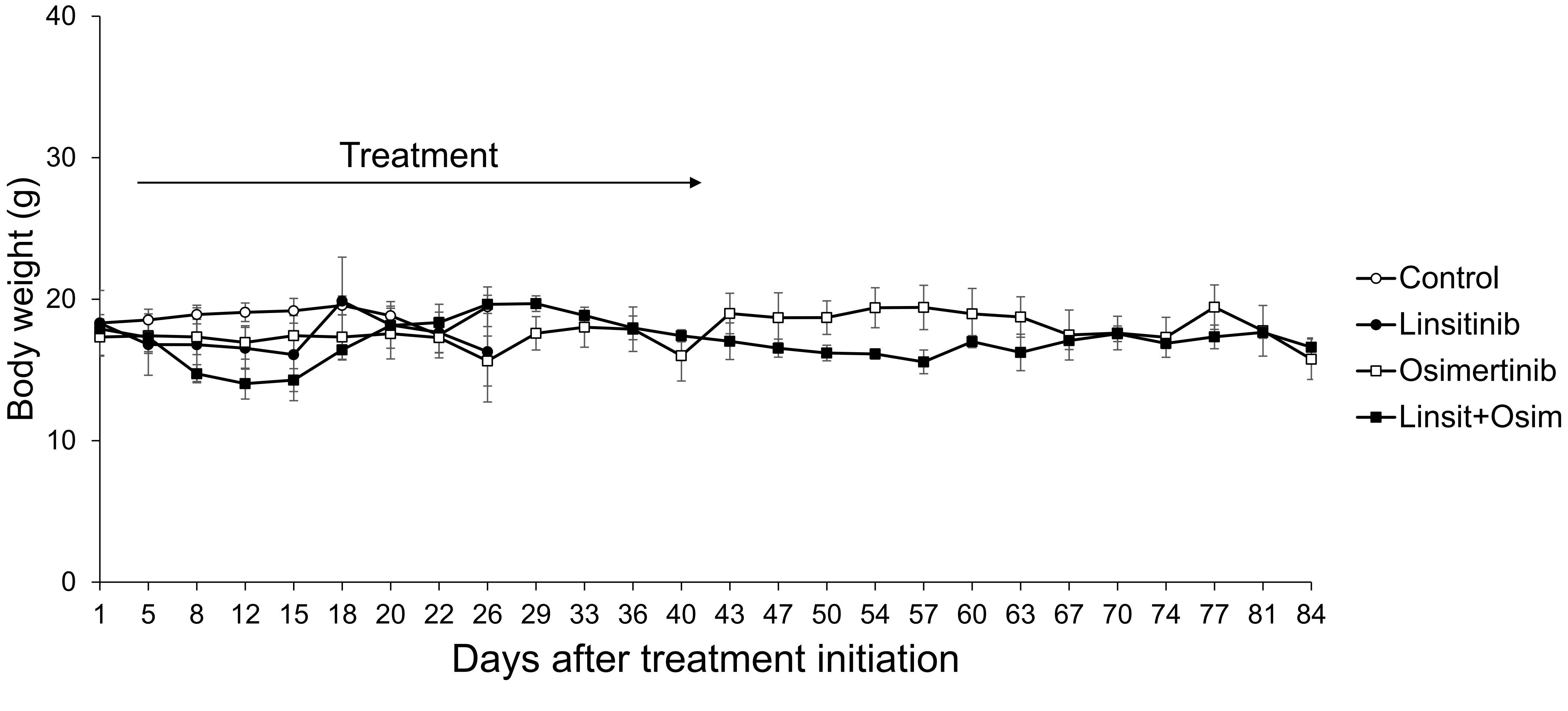

Supplementary Figure 14. Body weight of mice. Mice body weight is presented in Figure 6 (**a**) and Figure 7b (**b**). Data are the mean ± s.d. of 6 mice in each group.

Supplementary Table 1.
List of shRNA oligos, qRT-PCT primer sets, and Chip-qPCR primer sets.

Supplementary Table 1A; shRNA oligos

**shBCL6**	
Forward	CCGGCCACAGTGACAAACCCTACAACTCGAGTTGTAGGGTTTGTCACTGTGGTTTTT
Reverse	AATTAAAAACCACAGTGACAAACCCTACAACTCGAGTTGTAGGGTTTGTCACTGTGG
**shCEBPA**	
Forward	CCGGGCAACTCTAGTATTTAGGATACTCGAGTATCCTAAATACTAGAGTTGCTTTTT
Reverse	AATTAAAAAGCAACTCTAGTATTTAGGATACTCGAGTATCCTAAATACTAGAGTTGC
**shFOXA1**	
#1 Forward	CCGGGAACACCTACATGACCATGAACTCGAGTTCATGGTCATGTAGGTGTTCTTTTTG
#1 Reverse	AATTCAAAAAGAACACCTACATGACCATGAACTCGAGTTCATGGTCATGTAGGTGTTC
#2 Forward	CCGGGCGAAGTTTAATGATCCACAACTCGAGTTGTGGATCATTAAACTTCGCTTTTTG
#2 Reverse	AATTCAAAAAGCGAAGTTTAATGATCCACAACTCGAGTTGTGGATCATTAAACTTCGC
#3 Forward	CCGGGCGTACTACCAAGGTGTGTATCTCGAGATACACACCTTGGTAGTACGCTTTTTG
#3 Reverse	AATTCAAAAAGCGTACTACCAAGGTGTGTATCTCGAGATACACACCTTGGTAGTACGC
**shNFE2**	
Forward	CCGGCTAAGCTTTGGTCTATAAAGTCTCGAGACTTTATAGACCAAAGCTTAGTTTTTG
Reverse	AATTCAAAAACTAAGCTTTGGTCTATAAAGTCTCGAGACTTTATAGACCAAAGCTTAG

Supplementary Table 2B; qRT-PCR primer sets

Gene	Forward	Reverse
AR	CAGCCTATTGCGAGAGAGCTG	GAAAGGATCTTGGGCACTTGC
ARNT	GGGAACCTCACTTCGTGGT	CTGGGAGGGAAACACCTG
ARNTL	TTGCTGAGGAAATCATGGAA	GGCGTACTCGTGATGTTCAA
BACH1	GGACACTCCTTGCCAAATGCAG	TGACCTGGTTCTGGGCTCTCAC
BCL6	GACTCTGAAGAGCCACCTGC	CTGGCTTTTGTGACGGAAAT
BCL11B	CCCAGAGGGAGCTCATCAC	ACTTGGCTCCTCTATCTCCAGAC
BCLAF1	GTCTGGGTCTGGTTCTGTTGG	TTCTGTGGTGCGATTGTCTTT
BCOR1	CGATGCCTATAGCGATGTGTT	TCCGAAAGCAGTAGCCAGTT
CDK9	TTCGGGGAGGTGTTCAAG	ATCTCCCGCAAGGCTGTAAT
CEBPA	TGGACAAGAACAGCAACGAGT	TTGTCACTGGTCAGCTCCAG
CHD8	TGAACTGTTTGGGAATGGAA	TGCTGCTCTCTGGTGCAATA
CREB1	TGCCACATTAGCCCAGGTA	CCATTGGGCAGCTGTACTAGA
CREBBP	ACCGGTGTAAGGAAAGGCTG	TCAGGTGTTGGGAAGATGGC
CTCF	GAAAGAAGATTCCTCTGACAGTGA	TCTGGCTCAGGTTCAATTTCTAC
DNMT3A	TTCTACCGCCTCCTGCATGAT	GCGAGATGTCCCTCTTGTCACTA
DPF2	GGCAGAGGAACAGGGAAGAT	TGCTCCATGGCATCTTTGTA
E2F1	TGCAGAGCAGATGGTTATGG	CTCAGGGCACAGGAAAACAT
E2F6	GCTCCAGCAGAAACCAGATT	CCTGCTCCACTTCACACAAA
EGR1	AGCCCTACGAGCACCTGAC	GGTTTGGCTGGGGTAACTG
ELF1	CATCACCAGAACAGCCTAAGAG	TTGTGTTTCCCTTTCCATCTTTG
EP300	TCTTCAGCACCATGGACAGT	GTTGCATACGAGGCCCATAG
ERG	CGCAGAGTTATCGTGCCAGCAGAT	CCATATTCTTTCACCGCCCACTCC
ESR1	TTGCTCCTAACTTGCTCTTGG	CGAGATGATGTAGCCAGCAG
FLI1	TACAACCTCCCACACCGACCA	TGTTATTGCCCCAAGCTCCTCT
FOXA1	AGGGCTGGATGGTTGTATTG	ACCGGGACGGAGGAGTAG
FOXA2	TGAACGGCATGAACACGTA	GCCCACGTACGACGACAT
FOXK1	CAGTTACCGCTTTGTGCAGAA	CGGCTTTGACTCATCCTTGG
FOXM1	GCAGCATCAAGCAAGAGATG	GACGCTGATGGTCTCGAAG
FOXO1	AAGGGTGACAGCAACAGCTC	TTCCTTCATTCTGCACACGA
FOXP1	GGGGCAGTATGGACAGTGGATGA	TTGAGAGGTGTGCAGTAGGCGTG
GATA3	ACTACGGAAACTCGGTCAGG	GGTAGGGATCCATGAAGCAG
GTF2B	AGCAACAAAAGATCCATCTCG	AAAACTTGCAGCTCCTGTGC
HEY1	CGCCTCTGCTCTCCTCAG	GCTCAGTGCATTGGGAGAC
HIF1A	GATAGCAAGACTTTCCTCAGTCG	TGGCTCATATCCCATCAATTC
HMGB1	GCCTCCTTCGGCCTTCTT	ACAGGCCAGGATGTTCTCCTTT
IGF1R	GGCACAATTACTGCTCCAAAGAC	CAAGGCCCTTTCTCCCCAC
IRF1	AGGCTACATGCAGGACTTG	ACTGGGATGTGCCAGTCG
JUN	CGCCTGATAATCCAGTCCA	TTCTTGGGGCACAGGAACT
JUND	CACAGTTCCTCTACCCCAAGG	TTCTGCTTGTGTAAATCCTCCA
KDM2B	GAGGAGAAGAAGAAGGTGAAG	TTGATGGGCTGCTGGTTC
KDM5B	AAAAGCACCAAATTAGAGAGTCTGA	TTCCCCAAGAGTTGCCATAG
KLF1	ACACCAAGAGCTCCCACCT	GTAGTGGCGGGTCAGCTC
KMT2A	TCCTGAATACAACCCCAATGA	TGGCAGATCCATGCTAGTTG
KMT2D	AGGACCCCTTTGGACTGG	GCCCCCGTAGGACTAGGATA
MAX	TGTTGTTGTCGGTGACTTCC	CGTTATCGCTCATTTCCTACG
MAZ	AAGCGGTGAGGTTTGAGGA	CCCCCATACCACCTTATGAA
MED1	AACACCCTCATTGGAAGCTG	GGACACACTTCAAATTGGAGAA
MXI1	CCAGCTGCCACCTCTCCAT	TTGTAGGATAGAGGCTGTCATGCA
MYB	GACTATGATGGGCTGCTTCC	TGTTCCATTCTGTTCCACCA
MYC	CACCAGCAGCGACTCTGA	CTGTGAGGAGGTTTGCTGTG
MYCN	GATGCACCCCCACAGAAGAA	CTCCGAGTCAGAGTTTCGGG
MYOD1	CACTACAGCGGCGACTCC	TAGGCGCCTTCGTAGCAG

NFE2	GCTGTCCACTTCAGAGCTAGG	GCTCACTTGGAGCATTCAGA
NFKB1	ACCCTGACCTTGCCTATTTG	AGCTCTTTTTCCCGATCTCC
NFKB2	CACATGGGTGGAGGCTCT	ACTGGTAGGGGCTGTAGGC
NOTCH1	AGGCAATCCGAGGACTATGA	GCTCAGAACGCACTCGTTG
NR3C1	TCCCTGGTCGAACAGTTTTT	GCTGGATGGAGGAGAGCTTA
NRF1	CAGTCACTATGGCGCTTAACA	ATCTGTCCCCCACCTTGTAA
PAX5	TGTCCCCCATATCTGTATGTCA	GCTGATCCCAAGTCCAGTCT
RAG1	AATATCAACCAAATTGCAGACATC	GCCATGCTGGCTGAGGTA
RELA	ATGGCTTCTATGAGGCTGAGCT	CACACACTGGATTCCCAGGTT
RUNX1	TCAGGTTTGTCGGTCGAAG	GCCCATCCACTGTGATTTTG
RUNX2	ATCATCGCCGACCACCCGGC	GGCTACCACCTTGAAGGCCACG
SKI	CAGAGGAGGACAAGGACTCG	GAGGACAAGGAGGAGGTGAAT
SMARCC1	GGACTGAACAGGAGACCCTTC	CCGACACTTTGTTCCAATCA
SP1	GGACTGAACAGGAGACCCTTC	CCGACACTTTGTTCCAATCA
STAT1	CTTGTTGGGGCACAAGGT	CCATGGGAAAACTGTCATCA
STAT3	CCTGGTGTCTCCACTGGTCT	TCTGGCCGACAATACTTTCC
TAF1	AAGGATGCTGGCTATGGTGA	GTGTTCCAAGGGGCAGTG
TBX21	CCAACACGCATATCTTTACTTTCC	ACTCAAAGTTCTCCCGGAATC
TCF12	CCGTGGCAGTCATCCTTAGT	GCCGATACGGCAGAAACTT
TCF3	GGCACCCACTTCACTGAGTC	TCTCCCGAAGGAGGCATA
TFAP2A	AACATGCTCCTGGCTACAAAA	AGGGGAGATCGGTCCTGA
TFAP4	GAGGGCTCTGTAGCCTTGC	GAATCCCGCGTTGATGCTCT
TRIM28	TGGTCAATGATGCCCAGA	TTGGTCATGGTCCAGTGCT
YY1	GAAGCCCTTTCAGTGCACGTT	ACATAGGGCCTGTCTCCGGTAT
ZBTB7A	CATCTGCGAGAAGGTCATCC	TGTCCTGCCTGGTGAAGC
ZBTB17	TGGAGCTCAAGCCAGACC	GGCTCCACCTCCATTTCTT
ZNF143	CAACGACACTGAGCCCATC	TCTGGGACCCTTTAAGAACATC
ZNF384	CCCGAGATGAATGACCCTTA	AGTAGAATGTCAGTGAGCACATCC

Supplementary Table 1C; ChIP-qPCR primer sets

Region	Forward	Reverse
IGF1R Pro0	GGAGTCTCGCTTTGGGGAAA	AGTACCGACCCTAAGTCTCCGC
IGF1R Pro1	TCGGGCTTTCCAGTACGCAG	CGGTTCTTGAAAAGCTTCATTGTT
IGF1R Pro2	AACCCGAGGAGGAGCGAGC	GTGGGCAGGCTCGCTGG
