# Supplementary material for: Transient IGF-1R inhibition combined with osimertinib eradicates AXL-low expressing EGFR mutated lung cancer
Source: Nat Commun. 2020 Sep 14;11:4607. doi: 10.1038/s41467-020-18442-4 (PMC7490421; doi:10.1038/s41467-020-18442-4)
Supplement: Supplementary file 3 — Reporting Summary [file 41467_2020_18442_MOESM3_ESM.pdf]

nature research

Corresponding author(s): Seiji Yano

Last updated by author(s): Aug 20, 2020

Reporting Summary

Nature Research wishes to improve the reproducibility of the work that we publish. This form provides structure for consistency and transparency in reporting. For further information on Nature Research policies, see our Editorial Policies and the Editorial Policy Checklist.

Statistics

For all statistical analyses, confirm that the following items are present in the figure legend, table legend, main text, or Methods section.

n/a	Confirmed	
☐	☒	The exact sample size (*n*) for each experimental group/condition, given as a discrete number and unit of measurement
☐	☒	A statement on whether measurements were taken from distinct samples or whether the same sample was measured repeatedly
☐	☒	The statistical test(s) used AND whether they are one- or two-sided<br>*Only common tests should be described solely by name; describe more complex techniques in the Methods section.*
☐	☒	A description of all covariates tested
☐	☒	A description of any assumptions or corrections, such as tests of normality and adjustment for multiple comparisons
☐	☒	A full description of the statistical parameters including central tendency (e.g. means) or other basic estimates (e.g. regression coefficient) AND variation (e.g. standard deviation) or associated estimates of uncertainty (e.g. confidence intervals)
☐	☒	For null hypothesis testing, the test statistic (e.g. *F*, *t*, *r*) with confidence intervals, effect sizes, degrees of freedom and *P* value noted<br>*Give P values as exact values whenever suitable.*
☒	☐	For Bayesian analysis, information on the choice of priors and Markov chain Monte Carlo settings
☒	☐	For hierarchical and complex designs, identification of the appropriate level for tests and full reporting of outcomes
☒	☐	Estimates of effect sizes (e.g. Cohen's *d*, Pearson's *r*), indicating how they were calculated

Our web collection on statistics for biologists contains articles on many of the points above.

Software and code

Policy information about availability of computer code

Data collection	The data were collected from MTT assay, flow cytometry, tumor volume in vivo, IHC staining for tumors, and clinical responses rate.
Data analysis	GraphPad Prism8 was used for drawing the graphs of the cell viability.<br>BD FACS Diva was used for data collection and Flowjo Ver.10.5.3 for data analysis for flow cytometry.

For manuscripts utilizing custom algorithms or software that are central to the research but not yet described in published literature, software must be made available to editors and reviewers. We strongly encourage code deposition in a community repository (e.g. GitHub). See the Nature Research guidelines for submitting code & software for further information.

Data

Policy information about availability of data

All manuscripts must include a data availability statement. This statement should provide the following information, where applicable:

- Accession codes, unique identifiers, or web links for publicly available datasets
- A list of figures that have associated raw data
- A description of any restrictions on data availability

All relevant data are included in the paper and its supplementary information files "Source Data file". The source data for Figure 3I and Supplementary Figure 8A, 8B are available at https://chip-atlas.org/peak_browser.

Field-specific reporting

Please select the one below that is the best fit for your research. If you are not sure, read the appropriate sections before making your selection.

☒ Life sciences ☐ Behavioural & social sciences ☐ Ecological, evolutionary & environmental sciences

For a reference copy of the document with all sections, see nature.com/documents/nr-reporting-summary-flat.pdf

Life sciences study design

All studies must disclose on these points even when the disclosure is negative.

Sample size	No statistical methods were used to determine the sample size. Required sample sizes were determined based on previous experiments performed in our laboratory (Nat Commun 2019; doi:10.1038/s41467-018-08074-0.). For the determination of AXL- and phosphorylated IGF-1R expression, we collected clinical specimens as many as possible. This was the maximum sample size obtained by collaborating institutes in this study.
Data exclusions	No data were excluded.
Replication	Cell viability assay was performed with triplicate cultures and repeated at least three times with similar results.<br>Western blots with cell lines were performed three times with similar results..<br>Western blots with PDX were performed twice because of limited availability of samples.<br>IHC and H&E staining with in vivo tumors or clinical specimens was performed one time because of limited availability of samples.<br>Human tyrosine kinase array was performed one time.<br>For real-time qPCR, three biological replicates were performed, and each with three technical replicates.<br>All attempts at replication were successful.<br>In vivo experiments with tumor cell lines and PDX were performed one time with 4-6 mice/group and 2 mice/group, respectively.<br>FACS experiments were performed three times with similar results.<br>ELISA for IGF-1 and IGF-2 was evaluated one time with duplicate cultures.
Randomization	In vivo experiments, the mice bearing skin tumor were grouped (n=5 each group) randomly after the tumor establishment.<br>For in vitro experiments, randomization was not performed because tumor cells were allocated to the various conditions.
Blinding	All in vivo treatments were given as unblinded experiments.<br>All measurements for tumor size and data analysis were performed as blinding experiments.

Reporting for specific materials, systems and methods

We require information from authors about some types of materials, experimental systems and methods used in many studies. Here, indicate whether each material, system or method listed is relevant to your study. If you are not sure if a list item applies to your research, read the appropriate section before selecting a response.

Materials & experimental systems

n/a	Involved in the study
☐	☒ Antibodies
☐	☒ Eukaryotic cell lines
☒	☐ Palaeontology and archaeology
☐	☒ Animals and other organisms
☐	☒ Human research participants
☒	☐ Clinical data
☒	☐ Dual use research of concern

Methods

n/a	Involved in the study
☒	☐ ChIP-seq
☐	☒ Flow cytometry
☒	☐ MRI-based neuroimaging

Antibodies

Antibodies used	1.Phospho-Axl (Tyr702)(D12B2) Rabbit mAb :Cell Signaling Technology， cat number: #5724;<br>2.AHuman Axl Antibodyxl(C44G1) Rabbit mAb: Cell Signaling Technology， cat number: #4566S;<br>3.Anti EGFR(phospho Y1092) Antibody: abcam, cat number:ab40815;<br>4.Phospho-IGF-I Receptor β (Tyr1316) Antibody: Cell Signaling Technology , cat number:#28897;<br>5.IGF-1R antibody: Cell Signaling Technology, cat number: 9750s;<br>6.Phospho-Akt (Ser473) (D9E) XP® Rabbit mAb: Cell Signaling Technology, cat number:#4060L;<br>7.Akt Antibody(100μl): Cell Signaling Technology, cat number:#9272S;<br>8.Phospho-Gab1 (Tyr627) (C32H2)：Cell Signaling Technology, cat number:3233;<br>9.Gab1 Antibody: Cell Signaling Technology, cat number:3232s;<br>10.Shc Antibody: Cell Signaling Technology, cat number:2432s;<br>11. Phospho-Shc (Tyr317) Antibody: Cell Signaling Technology, cat number:2431s;

12.IRS-1 Antibody: Cell Signaling Technology, cat number:2382;
13.β-Actin(13E5) Rabbit mAb：Cell Signaling Technology, cat number:#4970S;
14.Phospho-p44/42 MAPK (Erk1/2) (Thr202/Tyr204)：Cell Signaling Technology, cat number:#4370S;
15.Anti-ERK1/ERK2: R&D SYSTEMS, cat number:AF1576;
16.Anti-EGF Receptor (D38B1) XP Rabbit mAb #4267：Cell Signaling Technology, cat number:4267s;
17. Rabbit Anti-FGFR3 (phospho Y724) antibody [EPR2281(3)]：abcam, cat number: (ab155960);
18.Rabbit Anti-FGFR3 antibody [EPR2304(3)]:abcam, cat number: (ab133644);
19.Brilliant Violet 421TM anti-human EGFR antibody (Biolegend Cat.No.352911);
20.FLEX Monoclonal Mouse Anti-Human Ki-67 Antigen[Clone MIB-1]:Dako,cat number: (F7268);
21. Human Axl Antibody:R&D systems,cat number: AF154;
22. Anti-rabbit IgG, HRP-linked Antibody:Cell Signaling Technology, cat number:7074;(secondary antibody)
23.Anti-mouse IgG, HRP-linked Antibody:Cell Signaling Technology, cat number: #7076;(secondary antibody)

Validation

W-Western IP-Immunoprecipitation IHC-Immunohistochemistry ChIP-Chromatin Immunoprecipitation IF-Immunofluorescence F-Flow Cytometry E-P-ELISA-Peptide
1.Phospho-Axl （Tyr702)(D12B2) Rabbit mAb :reactivity for human; application for WB;
2.AHuman Axl Antibodyxl(C44G1) Rabbit mAb: reactivity for human and Monkey; application for WB, IP and F;
3.Anti EGFR(phospho Y1092) Antibody: reactivity for Mouse, Human; application for WB, ICC/IF, IHC-P, Flow Cyt;
4.Phospho-IGF-I Receptor β (Tyr1316) Antibody: reactivity for human and Mouse; application for WB,IP;
5.IGF-1R antibody: reaçtivity for H M R Mk;application for WB, IP, IF and F;
6.Phospho-Akt (Ser473) (D9E) XP® Rabbit mAb: reactivity for H M R Hm Mk Dm Z B;application for WB, IP, IHC,IF and F;
7.Akt Antibody(100μl): reactivity for H M R Hm Mk C Dm B Dg Pg GP;application for WB, IP,IF and F;
8.Phospho-Gab1 (Tyr627) (C32H2)：reactivity for H ;application for WB;
9.Gab1 Antibody: reactivity for H M R Mk ;application for WB,IP;
10.Shc Antibody: reactivity for H M R ;application for WB;
11. Phospho-Shc (Tyr317) Antibody: reactivity for H M; application for WB;
12.IRS-1 Antibody: reactivity for H M R; application for WB IP;
13.β-Actin(13E5) Rabbit mAb:reactivity for H M R Mk B Pg; application for WB IHC IF F;
14.Phospho-p44/42 MAPK (Erk1/2) (Thr202/Tyr204):reactivity for H M R Hm Mk Mi Dm Z B Dg Pg Sc;application for WB IP IHC IF F ;
15.Anti-ERK1/ERK2: reactivity forHuman, Mouse, Rat;application for WB;
16.Anti-EGF Receptor (D38B1) XP Rabbit mAb: reactivity for H M Mk;application for WB IP IHC IF F;
17. Rabbit Anti-FGFR3 (phospho Y724) antibody [EPR2281(3)]:reactivity for Mouse, Human; application for Flow Cyt, Dot blot, WB, ICC/IF, IP;
18.Rabbit Anti-FGFR3 antibody [EPR2304(3)]:reactivity for Human;application for Flow Cyt,ICC/IF or IHC-P;
19.Brilliant Violet 421TM anti-human EGFR antibody :reactivity for Human;application for Flow Cyt,IF ;
20.FLEX Monoclonal Mouse Anti-Human Ki-67 Antigen[Clone MIB-1]:reactivity for Human;application for IHC, IHC-frozen;
21.Human Axl Antibody:R&D systems,cat number: reactivity for Human;application for IHC, IHC-P, IF, WB, ELISA;

Eukaryotic cell lines

Policy information about cell lines

Cell line source(s)

Six human NSCLC cell lines with mutations in EGFR were utilized.
The human NSCLC cell lines HCC4011 and H3255 were generously provided by Dr. David P. Carbone (Ohio State University Comprehensive Cancer Center, Columbus, OH) and Dr. John D. Minna (University of Texas Southwestern Medical Center, Dallas, TX), respectively. The human cell lines HCC827 and HCC4006 were purchased from the American Type Culture Collection (Manassas, VA), and the PC-9 cell line was obtained from the RIKEN Cell Bank (Ibaraki, Japan). The PC-9GXR cells, which contain deletions in the EGFR-exon 19 and the T790M mutation, were established at Kanazawa University (Kanazawa, Japan) from the PC-9 cell xenograft tumors in nude mice that had acquired resistance to gefitinib.
H3255 cells are not commercially available.

Authentication

All cell lines were authenticated by DNA fringerprinting.

Mycoplasma contamination

All cell lines were tested negative for mycoplasma contamination.

Commonly misidentified lines
(See ICLAC register)

No misidentified cell lines were used in our study.

Animals and other organisms

Policy information about studies involving animals; ARRIVE guidelines recommended for reporting animal research

Laboratory animals

Six-week-old SHO mice (Crlj:SHO-PrkdcscidHrhr) were obtained from Charles River (Yokohama, Japan). The mice were housed in a 12-h light:dark cycle at 23°C±2°C temperature with relative humidity of 50±20 %, and given ad-libitum access to food and water for the duration of the study. Mice were housed in specific-pathogen-free (SPF) conditions and cared for in accordance with Fundamental Guidelines for Proper Conduct of Animal Experiment and Related Activities in Academic Research Institutions under the jurisdiction of the Ministry of Education, Culture, Sports, Science and Technology of Japan.

Wild animals	Our study did not involve wild animals.
Field-collected samples	We did not use field-collected samples.
Ethics oversight	The study protocol was approved by the Ethics Committee on the Use of Laboratory Animals and the Advanced Science Research Center, Kanazawa University, Kanazawa, Japan (approval no. AP-122505).

Note that full information on the approval of the study protocol must also be provided in the manuscript.

Human research participants

Policy information about studies involving human research participants

Population characteristics	Specimens of tumors containing EGFR-activating mutations prior to the initial treatments with osimertinib as the 1st line treatment were obtained from 29 lung adenocarcinoma patients hospitalized at University Hospital, Kyoto Prefectural University of Medicine (Kyoto, Japan), Nagasaki University Hospital (Nagasaki, Japan), International Medical Center, Saitama Medical University (Saitama, Japan), or National Hospital Organization Kinki-chuo Chest Medical Center (Osaka, Japan).A total of 29 EGFR mutated NSCLC patients, treated with osimertinib between September 2018 and September 2019 were enrolled in this study. The sample characteristics included a median age of 72 years old (range: 44–88), with 22 female patients (75.9%), and 8 (27.6%) patients having a history of smoking. The histological subtypes were 28 adenocarcinoma (96.6%) and 1 not otherwise specified (NOS) (3.4%).
Recruitment	All patients were participants in Institutional Review Board of each Hospitals –approved studies. We cannot exclude potential sources of bias: self-selection bias might have been occured by the physicians when they decided to treat with osimertinib. As patients who had not treat with osimeritnib were not included in this study, this might have led to over- or under-estimation of the results.
Ethics oversight	All patients were participants in Institutional Review Board of Kyoto Prefectural University of Medicine, Nagasaki University, Saitama Medical University, and National Hospital Organization Kinki-chuo Chest Medical Center –approved studies and all provided written informed consent.

Note that full information on the approval of the study protocol must also be provided in the manuscript.

Flow Cytometry

Plots

Confirm that:

☒ The axis labels state the marker and fluorochrome used (e.g. CD4-FITC).

☒ The axis scales are clearly visible. Include numbers along axes only for bottom left plot of group (a 'group' is an analysis of identical markers).

☒ All plots are contour plots with outliers or pseudocolor plots.

☒ A numerical value for number of cells or percentage (with statistics) is provided.

Methodology

Sample preparation	For EGFR detection, single-cell suspensions of tumor cells (5×105) were treated on ice with or without Brilliant Violet 421TM anti-human EGFR antibody (Biolegend Cat.No.352911, 2.5⍰l/sample) for 30 min. For IGF-1R detection, single-cell suspensions of tumor cells (5×105) were pre-treated with PerFix-nc Kit (Beckman Coulter, Cat.No.B31167, 50 ⍰L 2% fetal bovine serum (FBS), and 5 ⍰L Buffer 1 per sample) at room temperature for 15 min for membrane permeabilization. After washing, resultant cells were incubated with 300 ⍰L of Buffer 2 and 1 ⍰L of IGF-1 Receptor B (D23H3) rabbit monoclonal antibody per sample, at room temperature for 30 min.
Instrument	BD FACSCANTOII was used for data collection.
Software	BD FACS Diva was used for data collection and Flowjo Ver.10.5.3 for data analysis.
Cell population abundance	5.0X10^5 cells were used for flow cytometry analysis.
Gating strategy	Tumor cell lines were gated on single cell gates followed by FSC-A/SSC-A tumor cells population.

☒ Tick this box to confirm that a figure exemplifying the gating strategy is provided in the Supplementary Information.
